# Oral motor and electromyographic characterization of adults with facial fractures: a comparison between different fracture severities

**DOI:** 10.6061/clinics/2017(05)04

**Published:** 2017-05

**Authors:** Amanda Pagliotto da Silva, Fernanda Chiarion Sassi, Endrigo Bastos, Nivaldo Alonso, Claudia Regina Furquim de Andrade

**Affiliations:** I Divisao de Miologia Orofacial, Hospital das Clinicas HCFMUSP, Faculdade de Medicina, Universidade de Sao Paulo, Sao Paulo, SP, BR; II Departamento de Fisioterapia, Fonoaudiologia e Terapia Ocupacional, Faculdade de Medicina (FMUSP), Universidade de Sao Paulo, Sao Paulo, SP, BR; III Divisao de Cirurgia Plastica, Hospital das Clinicas HCFMUSP, Faculdade de Medicina, Universidade de Sao Paulo, São Paulo, SP, BR

**Keywords:** Mandibular Fractures, Condylar Fractures, Open Reduction, Closed Reduction, Orofacial Functions

## Abstract

**OBJECTIVES:**

To characterize the oral motor system of adults with facial injuries and to compare the oral motor performance/function between two different groups.

**METHODS:**

An observational, descriptive, cross-sectional study was conducted in 38 patients presenting with facial trauma who were assigned to the Division of Orofacial Myology of a Brazilian School Hospital. Patients were divided into two groups: Group 1 (G1) consisted of 19 patients who were submitted to open reduction of at least one facial fracture, and Group 2 (G2) consisted of 19 individuals who were submitted to closed fracture reduction with maxillomandibular fixation. For comparison purposes, a group of 19 healthy volunteers was recruited. All participants underwent a clinical assessment that included an oral motor evaluation, assessment of the mandibular range of motions, and electromyographic assessment of the masticatory muscles.

**RESULTS:**

Clinical assessment of the oral motor organs indicated that G1 and G2 presented deficits related to the posture, position, and mobility of the oral motor organs. Patients also presented limited mandibular ranges of movement. Deficits were greater for individuals in G1, especially for maximal incisor opening. Additionally, patients in G1 and G2 presented a similar electromyographic profile of the masticatory muscles (i.e., patients with facial fractures presented lower overall muscle activity and significant asymmetrical activity of the masseter muscle during maximum voluntary teeth clenching).

**CONCLUSION:**

Patients in G1 and G2 presented similar functional deficits after fracture treatment. The severity of facial fractures did not influence muscle function/performance 4 months after the correction of fractures.

## INTRODUCTION

The human face constitutes the first contact point in several human interactions; thus, injuries and/or mutilation of the facial structures may have a disastrous influence on the affected person 1. The literature has reported an increasing occurrence of facial injuries over the last four decades, especially as a consequence of the increase in motor vehicle accidents as well as in urban violence 2,3.

Facial fractures can have long-term consequences, both functionally and aesthetically. The management of fractures to the face remains a challenge to all health professionals involved in treatment and rehabilitation (i.e., oral and maxillofacial surgeons and multidisciplinary teams), demanding a high level of both skill and expertise 4. According to the literature, mandibular fractures are the most common type of facial bone fractures and are frequently accompanied by condylar fracture, with a frequency that ranges from 26% to 57% 2,5,6. The high incidence of mandibular condylar fracture is attributable to the binding of the mandibular ramus, which has a high stiffness, to the mandibular condyle head, which has a low stiffness 7.

The functions executed by the oral myofunctional system depend highly on the mandibular movements. These functions are responsible for intraoral space modifications and have a strong impact on masticatory function, swallowing, and speech patterns because they are responsible for enabling adequate movements of the tongue and other soft tissues (i.e., amplitude) inside the oral cavity 8. Post-traumatic ankylosis, for example, can arise from intra-capsular condylar fractures. Temporomandibular joint (TMJ) ankylosis is an extremely disabling affliction that causes problems with mastication, swallowing, speech, appearance, and hygiene 9,10. Studies that describe and evaluate the impact of condylar fractures on the functions performed by the oral myofunctional system are limited. However, it is known that mandibular movements are necessary to maintain the production of synovial fluid 9,10. Therefore, one would expect the limitation, or even the impossibility, of mouth opening to have severe consequences for the TMJ, not only with respect to maintenance of joint lubrication but also to the overall mandibular range of motion 11.

Studies that describe the functional characteristics of post-facial injuries are very scarce. While mandibular fractures have been extensively documented, little information has been presented regarding fractures of the maxilla and zygoma, even though these fractures can also have severe functional consequences 12-14. Salentijn et al. 13 reported that patients with zygoma fractures present better functional outcomes when not submitted to surgical procedures. Tabrizi et al. 14 found that patients with condylar fractures associated with contralateral mandibular fractures present more signs of temporomandibular disorders compared with patients with isolated unilateral fractures. The authors emphasize that the indirect impact of facial fractures on the TMJ should be considered as an etiological factor in the development of temporomandibular disorders.

The restoration of normal function and long-term stability are indispensable for the successful recovery of facial injuries. Both function and stability, however, may be compromised by inadequate or incorrect postoperative muscular rehabilitation after fracture reduction. Muscle atrophy, denervation, alteration of fiber types, myofibrosis, decreased muscle mass, morphological alterations of the condyle, and facial nerve disorders are biological consequences of fracture reduction, which can have profound clinical consequences 5,6,15.

Despite the adopted medical treatment (i.e., opened or closed fracture reduction), the literature recommends oral motor rehabilitation in patients who have suffered facial injuries 16. An efficient oral motor assessment, including assessment of the masticatory muscles, can shed light on the best rehabilitation procedure to restore oral motor functions 17. A clearer understanding of the patterns of facial injuries will also assist health care providers in planning and managing the treatment of traumatic facial injuries. Such information can also be used to guide the future funding of public health programs, as considerable resources are needed for rehabilitation (i.e., multidisciplinary teams), thus placing a large burden on the health care system 3.

This study was designed to characterize the oral motor system of adults with facial injuries and to compare the oral motor performance/function between two distinct groups: one submitted to open reduction of at least one facial fracture and the other submitted to closed reduction of the facial fracture with maxillomandibular fixation.

## METHODS

An observational, descriptive, cross-sectional study was performed in patients presenting with facial trauma who were assigned to the Division of Orofacial Myology of a large Brazilian School Hospital (Hospital das Clínicas) between December 2010 and September 2014 for assessment and treatment. The study design was approved by the Ethics Committee for the Analysis of Research Projects of the Institution (CAPPesq HCFMUSP no. 495.639). Prior to their enrollment, all participants were informed of the purpose and procedures of the study, after which time they all provided written informed consent.

Thirty-eight adults (i.e., age >18 years) with facial fractures were divided into two groups: Group 1 (G1) consisted of 19 patients who were submitted to open reduction of at least one facial fracture, and Group 2 (G2) consisted of 19 individuals who were submitted to closed fracture reduction with maxillomandibular fixation.

The presence of facial fractures was confirmed by the medical team of the Division of Cranio-Maxillofacial Surgery of the same hospital. In line with the basic principles of trauma surgery regarding the open or closed reduction of fractures, open reduction has been recognized to be best for patients presenting with post-fracture malocclusion, an angulation of fracture displacement above 30°, translocation greater than 4 mm, or lateral overriding 18. However, for moderately displaced condylar fractures or for fractures with no evident displacement or no risk of displacement, closed reduction with rigid or elastic maxillomandibular fixation is used. In this case, an arch bar is used for approximately two weeks.

For comparison purposes, a group of 19 healthy volunteers was recruited (Control Group [CG]). The inclusion criteria for this group were the following: age >18 years, absence of oral motor disorders/deficits, absence of alterations in the scapular region, complete permanent dentition (absence/extraction of the third molar was accepted), Skeletal Angle Class I facial pattern, and absence of malocclusion. Individuals were excluded if they reported having previous orthodontic treatment.

All groups were paired by age. Individuals with pre-existing surgical procedures involving the head and neck, communication or hearing deficits, neurological disorders of any type, and/or cognitive deficits were excluded.

### Oral motor clinical assessment

Participants underwent clinical oral motor assessment. Individuals were examined while sitting on a chair in a room with appropriate lighting. The Expanded Protocol of Orofacial Myofunctional Evaluation with Scores (OMES-E) was used for this assessment 19. This protocol was constructed based on previous models of evaluation, with the addition of numerical scales that reflect the physical characteristics and orofacial behaviors of the subjects. Its purpose is to evaluate the components of the stomatognathic system (lips, tongue, mandible, and cheeks) in terms of aspect/posture, mobility, and performance during swallowing and mastication. The maximum score is 230.

Participants were evaluated individually by visual inspection, and the evaluation was later complemented by the analysis of images recorded on a digital camera Sony DSC-W120 (Sony, Manaus, Brazil).

To evaluate data reliability, all participants were assessed by two experienced speech-language pathologists. The speech-language pathologists who assigned the OMES-E scores had successfully passed specific training tests. The Kappa coefficient was used to verify agreement between examiners for the overall scores. The obtained result indicated a high level of agreement (>0.859).

### Mandibular range of movement

The technique used to measure the mandibular range of movement was based on the methodology already published in the literature 20. With the use of a digital caliper (Digimess Pró-Fono Digital Caliper), the following measurements (in millimeters) were collected:

1. maximal incisor distance - we measured the distance between the incisive faces of the mandibular and maxillary central incisors;
2. right lateral excursion - we measured the horizontal distance from the mandibular central incisor to the maxillary central incisor after asking the individual to glide his/her mandible to the right. When there was a midline deviation (i.e., between the mandibular and maxillary central incisors), we used the pertinent adjustment;
3. left lateral excursion - the same procedure described above was performed to measure mandibular lateralization to the left;
4. protrusion - for this measurement, the patient was asked to glide the mandible forward. We then measured the horizontal overlap between the mandibular central incisors and the maxillary central incisors;
5. horizontal dental occlusion overlap - we measured the distance between the occlusal face of the maxillary central incisors and the distal face of the mandibular central incisors.

### Surface electromyography (sEMG) evaluation

Two muscle groups were examined in this study: the anterior temporal muscles and the masseter muscles.

All EMG recordings were performed using standard surface sensors (SDS500). We used the Miotool 400 (Miotec® Biomedical Equipment, Brazil) 4-channel computer-based system and disposable double electrodes (SDS 500 Ag/AgCl, contact surfaces with a 10-mm diameter). This EMG system has a wide bandpass filter, a bandwidth (RMS) of 20 to 500 Hz, and a 60-Hz notch filter. The system uses the Active Electrode technology, which is a compact sensor assembly that includes a miniaturized instrument preamplifier. Locating the amplifier at the electrode site allows artifacts to be canceled and the signal to be boosted before being transferred down the electrode cable (noise level <5 µV RMS). Each EMG record was full-wave and low-pass filtered. The computer program indicates the mean, standard deviation (SD), minimum, maximum, and range of muscle activity during each trial. Muscle activity (EMG) was quantified in microvolts (µV).

The interelectrode distance was 10 mm. Four sets of two bipolar pre-gelled stick-on surface electrodes were applied to the skin on each side of the face over the anterior temporal muscles and masseter muscles to record myoelectrical activity during specific tasks involving the masticatory muscles. This electrode arrangement included a third ground electrode positioned on the right wrist. Electrical impedance at the sites of electrode contact was reduced because the skin was scrubbed with alcohol gauze pads.

The electric activity of the above-mentioned muscles was assessed at the following times 21,22:

- Rest - Participants remained seated with their heads positioned horizontally according to the Frankfort plane. After the pairs of EMG electrodes were placed over the skin, each participant was instructed to remain quiet and relax for a period of 1 minute. Three separate recordings of the resting condition were made, with a duration of 30 seconds each;
- Maximum voluntary tooth clenching on cotton rolls (CR) - two, 10-mm-thick cotton rolls were positioned on the mandibular second premolars/first molars of each subject. Individuals were asked to clench as hard as possible and to maintain the same level of contraction for 5 s. During the test, individuals were verbally encouraged to perform at their best. All individuals repeated the test three times;
- Maximum voluntary tooth clenching (MVC) - Individuals were asked to clench as hard as possible and to maintain the same level of contraction for 5 s; during the test, individuals were verbally encouraged to perform at their best. All individuals repeated the test three times.

To avoid any fatigue effect, a rest period of at least 3 minutes was allowed between each test. Subjects were instructed that, during clenching, they should not feel additional muscular/articular pain.

### sEMG data analysis

Surface EMG traces were evaluated for onset of, peak, and offset of activity during clenching events. Onset was identified as the point of upward excursion of the sEMG trace from resting baseline that led to the clenching event. The peak was the highest amplitude point of the sEMG clenching trace. The offset was the point at which sEMG activity returned to baseline. Computer software calculated the mean value of the action potential during the movements (onset-peak-offset). To compare the results between participants, sEMG amplitude values were normalized relative to rest to provide evidence of possible differences.

To perform intragroup and multiple comparisons between the groups, the average sEMG amplitude obtained in all tooth clenching tasks was standardized against the activity of the resting task.

### sEMG data reliability

Because subjective judgment was used for sEMG measurements, interjudge reliability was estimated. To establish the interjudge reliability of the measurements used in the study, a second experienced staff member who was blinded to the original results measured the same parameters of 35 randomly selected samples from the 342 total clenching events. Intraclass correlation coefficients were high for all comparisons (range of lower 95% confidence interval [CI]=0.9157-0.9728), suggesting strong consistency between examiners.

### Data analysis

The collected data were analyzed using IBM SPSS Statistics 22.0. The distribution of the data was non-normal for all variables. For this reason, the analysis was performed using non-parametric tests. In addition to the descriptive analysis, paired comparisons between groups were performed using the Mann-Whitney U test and multiple comparisons between the groups were performed using the Kruskal-Wallis test. Dunn’s test was used for the post hoc pairwise analysis. The adopted significance level was 5% for all analyses.

## RESULTS

The patients in G1 included 2 females and 17 males, with a mean age of 32.4 years (±10.76); the patients in G2 included 2 females and 17 males, with a mean age of 32.2 years (±11.19); and the participants in the control group included 5 females and 14 males, with a mean age of 32.9 years (±13.15). The groups did not present a significant difference in age (*p*=0.961). Apart from age, we also controlled the time between the facial fracture reduction and the oral motor assessment. The statistical analysis indicated that G1 and G2 were assessed within the same time period (Table 1).

A classification of the location of the fractures was performed based on computed tomogram records obtained from patients’ medical files. Most of the patients presented with bilateral fractures in more than one location of the face. Patients in G1 presented more fractures of the mandible body, whereas patients in G2 presented more condylar and maxilla fractures. The mean number of fractures per patient was 2.22 in G1 and 2.05 in G2 (Table 2).

Table 3 presents the results of the multiple and pairwise comparisons for the scores obtained on the OMES-E. As expected, patients with facial fractures differed from the control group in all of the assessed parameters, with the exception of the static posture and position of the oral motor organs. Although not significant, the median obtained for the assessed parameters indicated that G1 presented higher scores for the mobility of the oral motor organs and higher overall scores on the clinical protocol compared with G2.

The three groups were compared with respect to differences in the mandibular ranges of motion (Table 4). Differences were observed for all of the parameters when comparing the groups with facial fractures with the control group. No significant differences were observed between G1 and G2. However, when considering the medians obtained for the assessed measurements, G2 presented a better mandibular range of motion compared with G1, especially regarding the maximal incisor distance.

The descriptive data related to the electromyographic assessment of the masticatory muscles are presented in Table 5. Individuals in the control group presented higher muscle activity in both tooth clenching tasks.

For the purpose of comparing the electromyographic data obtained for each group, the coefficient of asymmetry between both sides of the face during the clenching tasks was calculated for each pair of muscles: ratio of the side with lower temporal muscle activity/side with higher temporal muscle activity, and ratio of the side with lower masseter muscle activity/side with higher masseter muscle activity. The closer the ratio is to 1, the greater the symmetry in the muscle activity in that specific task.

Data analyses indicated that the groups differed only in maximum voluntary tooth clenching (i.e., MVC – see Table 6). A significant difference was observed between G1 and the control group for the masseter muscle. Looking closely at our results, it is important to highlight that the comparison between G2 and the control group for the same muscle during MVC yielded results near the threshold of statistical significance.

## DISCUSSION

The treatment of facial fractures, especially those involving the condyle, depends greatly on individual biological characteristics and on adaptations made by the masticatory system 11. Although these characteristics differ by a wide range among patients, understanding the mechanisms underlying the recovery of oral motor functions is essential for a successful rehabilitation program.

As expected, our results confirmed that patients with facial fractures present a poorer performance when executing movements with the oral motor organs and in swallowing and mastication. Although no studies have specifically investigated the oral myofunctional system in individuals with facial fractures, we can correlate our findings with the functional outcomes of patients submitted to orthognathic surgery. Studies related to orthognathic surgery have documented postsurgical decreases in muscular extensibility and strength, increases in muscular fatigability, hypomobility, and alterations of biomechanical efficiency and the length of the masticatory muscles as clinical consequences 23. Moreover, these signs are still reported 6 months after surgery in patients submitted to mandibular distraction, most likely as a result of muscle fiber regeneration reduction due to stretching during surgical procedures 24. All of these findings are possible explanations for our results.

Skeletal muscles are systematically targeted by growth promoters, such as growth hormone, and produce their own growth factors that act as regulators of muscle fiber hypertrophy and muscle volume 12. However, these mechanisms are still not completely understood for masticatory muscles in humans. The hypothesis is that certain types of facial fractures can overstretch the muscles and trigger processes that lead to muscle atrophy, thus causing alterations in the masseter muscle fibers. Sciote et al. 12 found a decrease in muscle activity and muscle fiber recruitment associated with a reduction in muscle volume in individuals with few occlusal contacts. In our study, patients in G1 showed alterations in dental occlusion that were corrected through surgery, but this was not true for patients in G2. Overall, although patients in G1 presented with more severe facial fractures, their oral motor performance in the clinical assessment was very similar to that of patients in G2.

Our study also revealed deficits in the mandibular range of motion. Again, both groups of patients with facial fractures presented similar performances (i.e., limited range of motion). According to previous studies 20, the expected values for mandibular movements in healthy individuals are the following: maximal incisor opening, between 40 and 60 mm; mandibular lateral excursions - between 7 and 11 mm (i.e., to each side); and mandibular protrusion, between 7 and 11 mm, with no distinction between gender and age group. When looking more closely at our results, patients submitted to open fracture reduction presented a greater limitation in all mandibular movements, particularly maximal incisor opening.

Mandibular function requires adaptation to a wide variety of factors associated with the stomatognathic system 25. Studies that have investigated the mobility of the mandible after different facial fracture treatments present conflicting results. A few reports have indicated that patients who were submitted to open reduction exhibit less discomfort, better regeneration of the condyle, and better mandibular movement during mouth opening compared with patients submitted to closed reduction 13,26. Nogami et al. 17 reported better functional results in patients with condylar fractures who had been submitted to arthrocentesis compared with those who underwent conventional closed reduction with maxillomandibular fixation. According to the study, the first group presented measurements of maximal incisor opening of approximately 40 mm 3 months after fracture treatment, and the second group reached the same result only after 6 months. Additionally, other studies have reported variations in maximal incisor opening, i.e., between 32 and 64 mm, in the presence of mandibular deviations and discomfort in patients submitted to open reduction 27.

Although maximal incisor opening movements have traditionally been used to evaluate the function of the TMJ, Schneider et al. 26 argued that mouth opening must be considered a less sensitive parameter than other mandibular movements because a higher rotational component may compensate for a deficit in the translational movement of the condyle in the glenoid fossa. In contrast, according to the authors, the active protrusion of the mandible seems to be a more sensitive marker for the translational movement of the condyle, which may have more significance for the functional abilities of the TMJ.

Studies on the electromyographic assessment of patients with facial fractures were not found in the current literature. Again, a few correlations can be made with the data obtained from patients who were submitted to orthognathic surgery. For more than two decades, surgeons have relied on parameters of mastication to evaluate the functional success of orthognathic surgery 28,29. Several parameters to quantify the function of the masticatory system have already been reported and include chewing efficiency, maximum bite force, electromyographic activity of the masticatory muscles, and the maximum range of mandibular motion 29,30.

Several studies have reported that individuals with dentofacial deformities, compared with individuals with normal dental occlusion, have lower EMG activity in the masticatory muscles 28,21,32, lower occlusal force 28,31, few occlusal contacts 28,32, and lower masticatory efficiency 32,33. In some studies, chewing efficiency improved after surgical correction but did not reach control values 33, while in others, improvement could not be shown 34,35. Long-term increases in maximum voluntary bite forces after surgery have been reported, but the effects of orthognathic surgery on the occlusal forces generated during mastication remain unknown 31.

We found that our groups with facial fractures presented a similar electromyographic profile for the masticatory muscles (i.e., patients with facial fractures presented lower overall muscle activity and significant asymmetrical activity of the masseter muscle during maximum voluntary tooth clenching). Thus, our results suggest that fracture severity had no influence on the recovery of muscle activation as measured by the electrical muscle potential. This finding is in agreement with studies that investigated the outcomes of orthognathic surgery. These studies detected no significant changes in maximum voluntary bite forces 12-18 months after surgery 28,33. In our study, muscle overstretching and postsurgical dental occlusion alterations are possible explanations for the asymmetrical activation of the masseter observed in patients submitted to closed fracture reduction. However, during open reduction, surgical manipulation of the soft tissues can lead to edema and scars, which in turn can have a negative impact on muscle performance 27.

Our study had some limitations, such as the heterogeneity of facial fractures and the time frame, namely, the duration between the facial fracture reduction and the oral motor assessment was less than 4 months. These factors may have caused the lack of difference between our groups of patients. Future studies will involve the follow-up of these patients and the comparison of functional performance after oral motor rehabilitation.

Patients with facial injuries presented significant deficits related to posture, position, mobility of the oral myofunctional structures, mastication, and swallowing compared with healthy controls. The severity of facial fractures did not have an influence on muscle function/performance 4 months after fracture correction.

## AUTHOR CONTRIBUTIONS

Silva AP was responsible for the data collection, analysis and interpretation of the results, and manuscript writing. Sassi FC was responsible for organizing and conducting the statistical analyses, interpretation of the results and writing the major portion of the manuscript. Bastos E and Alonso N were responsible for the data analyses, interpretation of the results and manuscript writing. Andrade CR was responsible for the research and experimental design, contributed to data analysis and manuscript preparation.

## Figures and Tables

**Table 1:** Time between fracture reduction and oral motor assessment (in days).

Group	Median	Interquartil interval	U	Z	*p*-value
G1	51.0	22.00 – 78.0	168.000	-0.365	0.715
G2	57.0	30.00 – 101.0			

Legend: G1=patients submitted to open fracture reduction; G2=patients submitted to closed fracture reduction with maxillomandibular fixation; Mann-Whitney test.

**Table 2:** Classification of fractures locations.

		RH (n)	LH (n)	Bilateral (n)	Total (n)
**G1**					
**Mandible**	**Symphysis**	0	0	4	4
	**Parasymphysis**	1	1	0	2
	**Body**	6	2	2	10
	**Ramus**	2	1	0	3
	**Subcondylar Area**	0	0	0	0
	**Coronoid Process**	0	0	1	1
	**Angle**	2	1	0	3
	**Condylar Process**	2	2	0	4
**Maxilla**		1	4	2	7
**Zygoma**		2	2	2	6
** Total number of fractures**		16	13	11	40
**G2**					
**Mandible**	**Symphysis**	0	0	4	4
	**Parasymphysis**	0	1	0	1
	**Body**	3	4	0	7
	**Ramus**	4	1	0	5
	**Subcondylar Area**	1	0	1	2
	**Coronoid Process**	0	0	0	0
	**Angle**	0	0	0	0
	**Condylar Process**	3	4	2	9
**Maxilla**		3	3	4	10
**Zygoma**		0	1	1	2
** Total number of fractures**		14	14	12	40

Legend: RH= right hemiface; LH = left hemiface; n = number of patients; G1= patients submitted to open fracture reduction; G2= patients submitted to closed fracture reduction with maxillomandibular fixation.

**Table 3:** Comparisons among groups for the results of the Expanded Protocol of Orofacial Myofunctional Evaluation with Scores (OMES-E).

	Group	Median	Inquartil interval	Multiple comparison	Pairwise comparison
**Aspect/Posture**	G1	53.0	49.0 – 54.0	X^2^=20.803 df=2 *p*<0.001*	G1 = G2 *p*=0.068
	G2	55.0	53.0 – 57.0		G1 ≠ CG *p*<0.001*
	CG	58.0	55.0 – 60.0		G2 = CG *p*=0.068
**Mobility**	G1	80.0	69.0 – 91.0	X^2^=16.997 gl=2 *p*<0.001*	G1 = G2 *p*=1.000
	G2	74.0	67.0 – 90.0		G1 ≠ CG *p*=0.002*
	CG	99.0	86.0 – 108.0		G2 ≠ CG *p*=0.001*
**Performance swallowing/mastication**	G1	34.0	29.0 – 40.0	X^2^=24.407 gl=2 *p*<0.001*	G1 = G2 *p*=1.000
	G2	36.0	30.0 – 39.0		G1 ≠ CG *p*<0.001*
	CG	45.0	43.0 – 48.0		G2 ≠ CG *p*<0.001*
**Total Score**	G1	172.0	148.0 – 176.0	X^2^=24.467 gl=2 *p*<0.001*	G1 = G2 *p*=1.000
	G2	168.0	153.0 – 181.0		G1 ≠ CG *p*<0.001*
	CG	204.0	183.0 – 214.0		G2 ≠ CG *p*<0.001*

Legend: OMES-E= Expanded Protocol of Orofacial Myofunctional Evaluation with Scores; G1= patients submitted to open fracture reduction; G2= patients submitted to closed fracture reduction with maxillomandibular fixation; CG= control group; df= degrees of freedom;

* = significant results (*p*<0.05); Kruskal-Wallis Dunn’s test.

**Table 4:** Comparisons among groups for the mandibular range of movement in millimeters.

	Group	Median (mm)	Inquartil interval	Multiple comparison	Pairwise comparison
Maximal incisor distance	G1	25.8	13.4 – 34.4	X^2^=29.895 df=2 *p*<0.001*	G1 = G2 *p*=0.887
	G2	31.6	25.6 – 39.8		G1 ≠ CG *p*<0.001*
	CG	48.8	45.0 – 57.7		G2 ≠ CG *p*<0.001*
Right lateral excursion	G1	4.6	2.0 – 5.5	X^2^=18.256 df=2 *p*<0.001*	G1 = G2 *p*=1.000
	G2	4.2	2.6 – 6.9		G1 ≠ CG *p*<0.001*
	CG	8.0	6.6 – 8.9		G2 ≠ CG *p*=0.004*
Left lateral excursion	G1	3.0	1.6 – 6.0	X^2^=14.649 df=2 *p*=0.001*	G1 = G2 *p*=1.000
	G2	4.6	2.3 – 6.1		G1 ≠ CG *p*=0.002*
	CG	7.7	6.4 – 8.0		G2 ≠ CG *p*=0.005*
Protrusion	G1	4.2	2.8 – 5.4	X^2^=11.193 df=2 *p*=0.004*	G1 = G2 *p*=1.000
	G2	4.3	2.4 – 6.8		G1 ≠ CG *p*=0.008*
	CG	6.7	5.7 – 7.3		G2 ≠ CG *p*=0.018*

Legend: mm=millimeters; G1= patients submitted to open fracture reduction; G2= patients submitted to closed fracture reduction with maxillomandibular fixation; CG= control group; df= degrees of freedom;

* = significant results (*p*<0.05); Kruskal-Wallis Dunn’s test.

**Table 5:** Electromyographic characterization of the temporal and masseter muscles.

Task	Group	Median (µV)	Interquartil Interval
**MVC – left temporal muscle**	G1	8.5	4.5 – 15.8
	G2	13.1	6.4 – 22.3
	CG	23.4	13.0 – 36.8
**MVC – right temporal muscle**	G1	8.5	5.7 – 14.8
	G2	15.0	6.9 – 29.2
	CG	22.6	14.4 – 30.3
**MVC – left masseter muscle**	G1	7.2	3.6 – 15.3
	G2	13.7	6.6 – 22.3
	CG	28.3	14.4 – 39.0
**MVC – right masseter muscle**	G1	7.2	3.9 – 11.1
	G2	9.6	4.1 – 21.7
	CG	28.2	17.2 – 39.7
**CR – left temporal muscle**	G1	7.5	2.8 – 10.5
	G2	11.1	6.1 – 18.5
	CG	25.4	15.9 – 31.0
**CR – right temporal muscle**	G1	6.5	4.7 – 10.5
	G2	12.7	6.6 – 23.8
	CG	17.6	10.0 – 24.8
**CR – left masseter muscle**	G1	6.6	3.7 – 11.1
	G2	11.1	7.0 – 21.7
	CG	27.3	19.1 – 34.9
**CR – right masseter muscle**	G1	5.6	3.7 – 9.3
	G2	11.6	5.1 – 17.9
	CG	27.7	16.9 – 30.9

Legend: µV = microvolts; MVC= maximum voluntary teeth clenching; CR= maximum voluntary teeth clenching on cotton rolls; G1= patients submitted to open fracture reduction; G2= patients submitted to closed fracture reduction with maxillomandibular fixation; CG= control group.

**Table 6:** Comparisons among groups for the coefficient of asymmetry.

Coefficient of asymmetry	Group	Median (µV)	Interquartil interval	Multiple comparison	Pairwise comparison
**MVC – temporal muscle**	G1	0.58	0.31 – 0.75	X^2^=10.223 gl=2 *p*=0.006*	G1 = G2 *p*=1.000
	G2	0.59	0.29 – 0.77		G1 ≠ CG *p*=0.010*
	CG	0.8	0.7 – 0.9		G2 ≠ CG *p*=0.031*
**MVC – masseter muscle**	G1	0.66	0.58 – 0.81	X^2^=18.221 gl=2 *p*<0.001*	G1 = G2 *p*=0.183
	G2	0.50	0.36 – 0.63		G1 ≠ CG *p*<0.001*
	CG	0.9	0.8 – 1.0		G2 = CG *p*=0.051**
**CR – temporal muscle**	G1	0.61	0.23 – 0.77	X^2^=3.775 gl=2 *p*=0.151	-
	G2	0.53	0.25 – 0.74		
	CG	0.7	0.6 – 0.8		
**CR – masseter muscle**	G1	0.53	0.41 – 0.86	X^2^=4.293 gl=2 *p*=0.117	-
	G2	0.6	0.4 – 0.8		
	CG	0.85	0.52 – 0.89		

Legend: µV = microvolts; MVC= maximum voluntary teeth clenching; CR= maximum voluntary teeth clenching on cotton rolls; G1= patients submitted to open fracture reduction; G2= patients submitted to closed fracture reduction with maxillomandibular fixation; CG= control group; df= degrees of freedom;

* = significant results (*p*<0.05); Kruskal-Wallis Dunn’s test.
